# Leukemia-Initiating Cells in T-Cell Acute Lymphoblastic Leukemia

**DOI:** 10.3389/fonc.2017.00218

**Published:** 2017-09-25

**Authors:** Shi Hao Tan, Fatima Carla Bertulfo, Takaomi Sanda

**Affiliations:** ^1^Cancer Science Institute of Singapore, National University of Singapore, Singapore, Singapore; ^2^Department of Medicine, Yong Loo Lin School of Medicine, National University of Singapore, Singapore, Singapore

**Keywords:** T-cell acute lymphoblastic leukemia, leukemia initiating cells, TAL1, NOTCH1, core regulatory circuit

## Abstract

T-cell acute lymphoblastic leukemia (T-ALL) is a hematological malignancy characterized by the clonal proliferation of immature T-cell precursors. T-ALL has many similar pathophysiological features to acute myeloid leukemia, which has been extensively studied in the establishment of the cancer stem cell (CSC) theory, but the CSC concept in T-ALL is still debatable. Although leukemia-initiating cells (LICs), which can generate leukemia in a xenograft setting, have been found in both human T-ALL patients and animal models, the nature and origin of LICs are largely unknown. In this review, we discuss recent studies on LICs in T-ALL and the potential mechanisms of LIC emergence in this disease. We focus on the oncogenic transcription factors *TAL1, LMO2*, and *NOTCH1* and highlight the significance of the transcriptional regulatory programs in normal hematopoietic stem cells and T-ALL.

## Introduction

Since the establishment of functional repopulation assays in the late 1990s, accumulating studies have demonstrated the existence of cancer stem cells (CSCs) that possess self-renewal capability and the potential to generate differentiated daughter cells (1–4). Purification of a unique cell population based on the expression of specific cell surface markers enabled the prospective isolation of CSCs in various types of cancers. A prime example is acute myeloid leukemia (AML), which has been extensively studied as a model disease for the establishment of the CSC theory. Although T-cell acute lymphoblastic leukemia (T-ALL) has many similarities in pathophysiological features to AML, the CSC concept in T-ALL has not been firmly established. Leukemia-initiating cells (LICs), which can generate leukemia in a xenograft setting, have been confirmed in both human T-ALL patients and mouse models (5–12), but common stem cell markers have not been identified in this disease.

Unlike AML, which arises from the bone marrow, T-ALL clones originally emerge in the thymus, which does not provide a niche for hematopoietic stem cells (HSCs) (13–15). In many T-ALL cases, oncogenes are driven by a chromosomal translocation involving the *T-cell receptor* (*TCR*) gene locus, which is associated with somatic recombination in immature thymocytes (16). This suggests that T-ALL arises from committed T-cell precursors, but not from multi-potent HSCs. It is likely that developing thymocytes acquire stemness capability as a consequence of genetic and epigenetic abnormalities. On the other hand, recent studies have shown that early thymocytes can self-renew under certain condition (17, 18). Therefore, it is also possible that T-ALL arises from thymocytes that already possess self-renewal potential.

## Genetic Abnormalities in T-ALL: Overview

Acute lymphoblastic leukemia (ALL) is the most common type of childhood malignancy (19). Approximately 20% of ALL cases are classified as T-ALL. T-ALL is an aggressive malignancy characterized by the clonal proliferation of immature T-cell precursors that arise from the thymus and infiltrate into the bone marrow and peripheral blood (13–16). Enormous progress has been made in the treatment of T-ALL in the past few decades, with long-term remission observed in approximately 80% of children and 60% of adult patients (20, 21). However, a substantial fraction of T-ALL patients fail to respond to induction therapy or relapse within 2 years of diagnosis. The prognosis for this group of patients is very poor, with a 5-year survival rate of less than 25% (22).

T-ALL development requires multi-step genetic alterations of crucial oncogenes and tumor suppressors *via* different recurrent mechanisms, such as chromosomal translocations, intrachromosomal rearrangements, and mutations in protein-coding genes or enhancer elements, as well as epigenetic abnormalities (13–16). These alterations commonly affect genes that are required for cell growth, survival, and differentiation during normal T-cell development (14, 16). Results from recent genome-wide sequencing studies across different types of cancers indicate that ALL exhibits the fewest genomic abnormalities compared with other hematological malignancies and solid tumors (23, 24). This suggests that relatively few molecular alterations are crucial and significant enough to hijack the normal developmental program and promote malignant transformation.

### Molecular Abnormalities That Delineate the T-ALL Subgroups

Chromosomal translocation is a hallmark of T-ALL (16, 25). The most commonly observed translocations involve the *TCR* loci on chromosome 14q11.2 (*TCR alpha/delta*), 7q34 (*TCR beta*), and 7p14 (*TCR gamma*). They are often fused to a range of oncogenic transcription factors that are important during different stages of normal hematopoiesis and lymphocyte development (13–16), resulting in constitutive and ectopic expression of these factors. The affected genes include transcription factors from the basic helix-loop-helix family, including *TAL1, TAL2*, and *LYL1*; the homeobox family, including *TLX1, TLX3*; the *HOXA* genes; *NKX2-1*; *MYB*; and the LIM domain-only (LMO) genes *LMO1* and *LMO2*.

Cytogenetic analysis coupled with gene expression profiling has been used to classify T-ALL into several subgroups: *TAL1/LMO1/2-, TLX1/3-, HOXA/MEISI-, LMO2/LYL1*, and *NKX2-1-*positive T-ALL cases (25–27). Briefly, *TAL1, LMO2*, and *LYL1* are essential regulators of hematopoiesis (28–33). Those factors can be oncogenic when abnormally or ectopically overexpressed in immature T-cells (8, 34, 35), as we discuss later. Besides translocation, *TAL1* is aberrantly induced by intrachromosomal rearrangement or mutations in the enhancer (36–38). *TLX* genes are expressed during embryogenesis and required for normal development of the spleen (39). Overexpression of *TLX1* leads to T-ALL and exhibits aneuploidy in a mouse model (40). The *HOX* genes are a family of homeodomain containing transcription factors, which are expressed in HSCs and immature progenitors compartments (41). HOX cofactors such as MEIS1 which is important to improve binding selectivity and specificity of HOX proteins are also found to be overexpressed in T-ALL (42). Notably, these subgroups are mutually exclusive to each other and reflect the arrest of T-cell differentiation at different stages, including (a) early blockage at the CD4^−^CD8^−^ double-negative (DN) stage of thymocyte development for the *LMO2/LYL1* group, (b) early cortical T-ALL (CD1a^+^, CD4^+^, and CD8^+^) with expression of *TLX1/3 or NKX2-1*, and (c) late cortical T-ALL (CD3^+^, CD4^+^, and CD8^+^) with expression of *TAL1* (26, 43). More recently, the early T-cell precursor (ETP) subtype has been defined based on cell surface markers and gene expression profiles (43). ETP is enriched in the *LMO2/LYL1* group but can be also found in other subgroups (27).

### Activation of the NOTCH1 Pathway

Another major molecular abnormality in T-ALL is the mutations that affect the *NOTCH1* pathway (13–16). *NOTCH1* signaling is essential for normal T-cell precursor development and is strictly regulated in a ligand-dependent manner. Remarkably, activating mutations affecting *NOTCH1* are observed in more than 50% of T-ALL cases (44). Aberrant activation of *NOTCH1* was originally identified in T-ALL cases harboring the t(7;9)(q34;q34.3) chromosomal translocation, through which the intracellular form of NOTCH1 (ICN1) gene fuses to the *TCR beta* regulatory element, leading to expression of a constitutively active, truncated form of NOTCH1 (45). However, the majority of aberrant *NOTCH1* activation observed in T-ALL occurs due to mutations in its heterodimerization (HD) domain and/or the PEST domain (44). Mutations in the HD domain cause the NOTCH1 receptor to be susceptible to proteolytic cleavage and release of the ICN1 protein, while the PEST domain mutations inhibit the proteasomal degradation of ICN1 by the FBXW7 ubiquitin ligase, thus lengthening its half-life in T-ALL cells. Additionally, deletions or inactivating mutations of *FBXW7* are frequently observed in T-ALL (46, 47).

The oncogenic roles of NOTCH1 signaling in T-ALL have been extensively studied both in humans and in animal models. Overexpression of ICN1 protein in mouse hematopoietic progenitor cells leads to very rapid onset of T-ALL (48). Subsequent studies have identified the direct transcriptional targets of NOTCH1 in T-ALL, which are enriched in genes responsible for cell proliferation, metabolism, and protein synthesis, including *MYC* and *HES1* (49–53). These studies implicated *NOTCH1* as a driver oncogene in T-ALL.

### Epigenetic Regulators and Other Molecular Abnormalities

Alterations in genes that encode for epigenetic regulators such as *EZH2, SUZ12*, and *EED* have been also identified in T-ALL (54–57). These genes make up the core components of the polycomb repressor complex 2 that mediates the repressive histone mark H3 lysine 27 trimethylation (H3K27me3). Loss-of function mutations in these genes can lead to accelerated leukemia onset in mice (54, 55), suggesting that they act as tumor suppressors in T-ALL. Recent studies have shown that the KDM6A/UTX, which is responsible for demethylating H3K27me3, have cases of inactivating lesions and downregulation of this gene accelerates NOTCH1-driven leukemia in mice (55, 56). In contrast, another study showed that KDM6A/UTX acts as a pro-oncogenic cofactor when it is recruited by TAL1 in T-ALL to activate target gene expressions (57).

Other recurrent molecular abnormalities include genes that encode for proteins involved in the JAK-STAT signaling pathway, such as *IL7R, JAK1, JAK3*, and *STAT5B*; genes that are involved in PI3K-AKT signaling pathways, such as *PI3K* and *PTEN*; and genes involved in RAS-MAPK signaling pathways, such as *HRAS, KRAS*, and *PTPN11* (13–16). Additionally, recent sequencing studies discovered several new alternations including mutations in *CCND3, CTCF*, and *MYB* genes (27), and *SPI1/PU.1* fusions (58).

## CSC and LIC Concepts

The concept of CSCs originates from the observation that tumors consist of a hierarchically organized, heterogeneous population of cells with a minority of biologically distinct subsets capable of self-renewing and giving rise to clonal daughter cells (1–4). A number of studies have shown the existence of CSCs in various types of cancers. The CSC model also indicates that this rare cell population is able to tolerate therapeutic agents such as chemotherapy and radiation that eradicate the bulk of the rapidly proliferating tumor cells, thus resulting in inevitable cancer relapse in the long term (1–4, 59).

The most definitive property of stem cells lies in their self-renewal ability (1–4). Self-renewal in normal cells or CSCs gives rise either to one stem and one differentiated daughter cell *via* asymmetric division or to two stem cells *via* symmetric division. The general consensus in stem cell research is that CSCs are able to initiate and maintain clonal growth in long-term repopulation assays where the cancer cells are serially transplanted into immunodeficient recipient mice. The purification of a unique cell population based on the expression of specific cell surface markers has allowed researchers to isolate CSCs in various cancers, including AML and breast cancer (60–62). However, such populations have not been well characterized in many other cancers, including T-ALL. Hence, other terms, such as “tumor-initiating cells (TICs)” or “LICs,” have been coined to refer to the ability of transplanted cells to initiate tumor formation or leukemia in animals and are more preferentially used in experimental settings (1). Notably, the TIC/LIC concept is distinct from the “cell-of-origin” idea, as TIC/LIC strictly refers to cells in which tumorigenesis can be initiated (63), whereas the cell of origin that received the first oncogenic “hit” would progressively accumulate mutations during clonal evolution of the tumor. The acquisition of stem cell-like properties may occur at a much later stage of tumorigenesis in the evolved cells than the original cell that received only the first hit. In this regard, John Dick has proposed that TICs/LICs should be defined by their ability to (a) generate tumors in xenograft models that are representative of the parent tumors, (b) generate tumors upon serial passages in xenograft models, and, lastly, (c) give rise to daughter cells that can proliferate but might not be able to establish tumors after serial passages (1).

## LICs in Human AML and ALL: Discovery and Challenges

The presence of LICs was first reported by Dick and his colleagues in the late 1990s in studies of AML (60, 61). In a series of seminal studies, they showed that a rare subset of CD34^+^CD38^−^ cells isolated from AML patients was able to initiate the disease when transplanted into severe combined immunodeficient (SCID) mice (60). Crucially, the more differentiated CD34^+^CD38^+^ cells were unable to generate leukemia. In the initial study, secondary transplant of leukemic cells from SCID mice failed to generate leukemia. However, using a more immunocompromised non-obese diabetic (NOD)/SCID mouse model, the authors demonstrated that CD34^+^CD38^−^ cells have self-renewal properties (61). Furthermore, this group has shown that the engrafted CD34^+^CD38^−^ cells were able to give rise to more differentiated leukemic cells (61). Thus, this study demonstrated the presence of a leukemic hierarchy, with the CD34^+^CD38^−^ LICs at the top of the pyramid.

These results have also been challenged by studies utilizing more immunocompromised mouse models. For example, in the NOD/LtSz-scid IL-2Rγchain^null^ (NSG) mouse model, AML LICs are not only present exclusively in CD34^+^CD38^−^ cells (64). Results from this model showed that LICs can also be found in more differentiated CD34^−^ and CD38^+^ cells. The concept of LICs was also challenged by a study in which leukemic cells from *Ras*-induced T-cell lymphoma or an *E*μ*-Myc* model of pre-B/B-cell lymphoma were shown to engraft in non-congenic animals regardless of the number of cells injected (65). The authors stressed the need to interpret data from serial transplantations more carefully, since failure to show engraftment could simply be due to the inability of the human cells to adapt to the microenvironment in the mouse.

The identification of LICs in ALL is even more challenging. To date, the identity and presence of LICs in human ALL has not been firmly established and is still debatable. Early studies in B-cell ALL (B-ALL) reported that the relatively immature CD34^+^CD19^−^ cells could contain LICs (66, 67). However, recent studies have found that more mature CD34^+^CD19^+^ leukemic blasts could initiate leukemia in *ETV6-RUNX1-* or *TEL-AML1*-positive B-ALL cases (68). In addition, a more recent study on *MLL-AF4*-positive infant ALL indicated that the LICs capable of reconstituting transplanted mice are exclusively CD19^+^ but exhibit variable CD34 expression (69). These studies highlight the heterogeneity of LICs in B-ALL cases and suggest that different cytogenetic abnormalities might play a role in determining the type of LICs present.

Similarly, the nature of LICs in human T-ALL has not been well characterized. An early study suggested that CD34^+^CD4^−^ and CD34^+^CD7^−^ cells, which make up a fraction of the leukemic cells from pediatric T-ALL patients, had leukemia-initiating properties when engrafted into NOD/SCID mice (5). A follow-up study investigating LIC activity in cortical/mature T-ALL patients reported that the CD34^+^CD7^−^ population from these patients contained normal hematopoietic cells that were able to differentiate into different lineages, while the CD34^+^CD7^+^ cells possessed LIC capability (6). Dick and Chiu et al. have also reported that the CD7^+^CD1a^−^ subset is enriched for LIC activity and exhibits glucocorticoid resistance (7).

## LICs in Animal Models of T-ALL

Although the findings on LICs in primary human T-ALL are limited, several studies have been performed on transgenic animal models of T-ALL.

### LICs in the Tal1-Induced Mouse Models of T-ALL

One of the most commonly used T-ALL mouse models in the study of LICs is the *Tal1* transgenic mouse model; approximately 30% of these mice develop leukemia after a long latency period (8, 34, 35). Notably, tumor onset and progression can be accelerated by co-expressing the oncogene *Lmo1* or *Lmo2*. Tremblay and Hoang et al. have found that overexpression of *Tal1* and *Lmo1* resulted in a marked expansion of T-cells making up the CD4^−^CD8^−^ DN1, DN3, and DN4 populations and blocked differentiation into the CD4^+^CD8^+^ double-positive (DP) stage (8). The leukemia cells contain LICs that can generate leukemia in transplanted mice. Interestingly, they demonstrated that LICs are enriched in the DN population, especially DN3 and DN4, compared with the DP population and that these LICs could give rise to more differentiated leukemic cells (8). This study suggested that committed DN-stage T-cells with ectopic expression of *Tal1* and *Lmo1* exhibit self-renewal properties while retaining the potential to differentiate. A subsequent study by Kelliher and her colleagues utilizing the *Tal1/Lmo2* mouse model of T-ALL also showed that the DN3 and DN4 populations of leukemia cells possess LIC properties and drive T-ALL leukemogenesis (9, 10). In support of these data in double transgenic mice, McCormack and Curtis et al. demonstrated that *Lmo2* single transgenic mice show an increase in thymic progenitors in the DN3 subset while also displaying properties of LICs in serial transplantation experiments (11). Interestingly, several genes, such as *Hhex* and *Lyl1*, that are normally expressed in HSCs were expressed in the self-renewing cells. This suggests that an HSC-like transcriptional program might be induced in T-ALL cells. Taken together, these studies indicated that DN3 thymocytes gained self-renewal potential.

### Significance of NOTCH1 Activation in Mouse Models of T-ALL

Notably, gain-of-function mutations of the *Notch1* gene are frequently found in the *Tal1*/*Lmo1* mouse model of T-ALL (8, 70), similar to observations in human T-ALL (44). Tremblay and Hoang et al. reported that *Notch1* mutations occurred mostly at the DN4 preleukemic stage and that the mutations could also be observed during overt leukemia in the same mice (8). Interestingly, leukemia development and *Notch1* mutations were abolished in the absence of *CD3e*. Similarly, Cui and Mackall have reported that forced expression of *TCR* during early stages of T-cell development caused T-ALL in 100% and all cases harbored *Notch1* mutations (71). These results suggested that pre-TCR and TCR signaling have a permissive role in the acquisition of *Notch1* mutations and that active NOTCH1 signaling confers clonal dominance upon leukemia development.

Importantly, Tremblay and Hoang et al. showed that *Notch1/Tal1/Lmo1* triple transgenic mice developed leukemia significantly faster than single or double transgenic animals (8). The DN1–DN2 and DN3–DN4 subsets from *Notch1/Tal1/Lmo1* triple transgenic mice were able to induce T-ALL in secondary hosts with high efficiency compared with *Tal1/Lmo1* double transgenic mice (8). A subsequent study from the same group further suggested that *Notch1* drives self-renewal of thymocytes from the *Tal1/Lmo1* mouse model *via* its target genes *Hes1* and *Myc* (12). Treatment of the leukemic cells before and throughout the transplantation period with γ-secretase inhibitor, which inhibits the catalytic cleavage of NOTCH1, completely abolished the LIC function of the leukemic T-ALL cells. Given the importance of active NOTCH1 signaling in primary human T-ALL patient samples, these studies support the hypothesis that *Notch1*-activating mutations are important for the cells to gain clonal dominance during disease development.

Notably, a recent study by Pear and his colleagues showed that LICs in T-ALL induced by the overexpression of a mutant form of *NOTCH1* in adult mouse bone marrow progenitor cells are enriched in a single-positive (SP) T-cell population consisting of the CD8^+^CD4^−^HSA^hi^ fraction of cells (72). Thus, the types of LICs generated could be different from those found in the *Tal1*/*Lmo* transgenic mouse model.

### LICs in Other Animal Models of T-ALL

Additionally, several other animal models of T-ALL have been used to analyze LICs. In studies of *Pten*-null mice, which develop T-ALL with 100% penetrance, LICs are identified as cKit^mid^CD3^+^ cells and often overexpress *Myc* due to a recurrent chromosomal translocation at t(14;15). The self-renewal properties of these LICs could also be abolished *via* targeting both the deregulated PI3K signaling pathway and *Myc* expression concurrently (73, 74).

Apart from studies in T-ALL mouse models, a T-ALL zebrafish model has also been employed to investigate the presence of LICs in T-ALL. Langenau and Look et al. reported that the *Myc*-induced T-ALL zebrafish model demonstrates very similar molecular characteristics to human T-ALL patients that overexpress *TAL1* and *LMO2* (75). More recently, Langenau and his colleagues used syngeneic clonal zebrafish that can be transplanted into hosts without prior irradiation to show that the proportion of LICs in the *Myc*-induced T-ALL zebrafish model is much higher than previously reported (76). Further studies by the same group demonstrated that abnormal activation of the AKT-mTORC1 signaling pathway is the main underlying cause of the acquisition of LIC potential (77). These results support the mouse studies on LICs in T-ALL.

## The Role of Microenvironment in T-ALL Pathogenesis

Another important consideration in the study of LICs is the interaction between leukemia cells and non-leukemia cells in the microenvironment. Bone marrow niche is essential for the maintenance and regulation of normal HSCs (78, 79). AML and ALL cells also home and expand in the bone marrow. Several studies have shown that signals from the bone marrow niche can dictate the survival of LICs and their responses to various types of treatment administered (80, 81).

Notably, two recent studies have elucidated the roles of bone marrow niche in T-ALL pathogenesis and implicated the CXCL12-CXCR4 signaling axis in the maintenance and progression of T-ALL (82, 83). CXCL12 is a chemokine secreted from endothelial and mesenchymal cells in the bone marrow and binds to its G protein-coupled receptor CXCR4 (79). Pitt et al. showed that in the bone marrow, T-ALL cells reside in close contact with stroma cells that secrete Cxcl12 (82). Deletion of the *Cxcr4* receptor resulted in a reduction of leukemia burden and their infiltration into the bone marrow, thymus, and spleen in mouse model of T-ALL (82). Treatment of patient-derived human T-ALL cells in xenografts with a CXCR4 antagonist also produced the same result. Importantly, the authors observed a reduction in LIC activity in the absence of *Cxcr4* in mice (82). Passaro et al. independently showed that depletion of *CXCR4* affected T-ALL cell migration and expansion (83). Furthermore, the authors reported that calcineurin regulates CXCR4 expression in a cortactin-dependent manner (83). Those studies demonstrated the roles of the bone marrow niche in the maintenance of T-ALL.

## Self-Renewal Capability of T-ALL Cells: Does it Already Exist in the Thymus or is it Acquired?

One of the fundamental questions in LIC research is whether the LICs are derived from cells that already have self-renewal potential, such as HSCs, or whether they emerge from differentiated cells by newly acquiring stemness capability. T-ALL is derived from committed T-cell precursors in the thymus, which does not provide a niche for HSCs. The chromosomal translocation involving the *TCR* gene locus found in many T-ALL cases is associated with somatic recombination in immature thymocytes (16). These findings suggest that developing thymocytes likely acquire stemness capability as a consequence of genetic and epigenetic abnormalities. *Tal1* and *Lmo1/2* transgenic mice show an increased number of thymic progenitors that can generate leukemia, indicating that these oncogenic transcription factors are capable of inducing LIC ability in immature thymocytes.

On the other hand, recent studies have shown that normal thymocytes can self-renew in the absence of competitive precursor replacement (17, 18, 84). In general, HSCs differentiate into common lymphoid progenitor (CLP) cells in the bone marrow. CLPs migrate into the thymus and are committed to T-cell precursors that can differentiate into the DN to DP stage of thymocytes. In this well-accepted model, a continuous supply of lymphoid progenitor cells from the bone marrow is necessary to support T-cell development. Interestingly, Martins and Rodewald et al. recently reported that in *Rag2*^−^*^/^*^−^*γc*^−^*^/^*^−^*Kit^W/Wv^* mice, which do not produce lymphoid progenitors from the bone marrow, a transplanted wild-type thymus sustained T-cell development for a long period of time (17). Similarly, Peaudecerf and Rocha et al. reported that in *Rag2*^−^*^/^*^−^*γc*^−^*^/^*^−^*IL7 receptor*^−^*^/^*^−^ mice engrafted with a wild-type thymus, persistent development of donor T-cells was observed (18). In this setting, host lymphoid progenitors can still migrate into the thymus and replace donor thymocytes but cannot differentiate after the DN2 stage, because IL7R signaling is required for the proliferation of early T-cell progenitors. Thus, competitive replacement by the host lymphoid progenitors is restricted to the DN1 and DN2 stages in this mouse model. This indicates that the donor thymus, which contains DN3 thymocytes, sustained T-cell development. Although this mechanism may be activated only when the competitive DN3 thymocytes are absent, these studies indicate that the thymus harbors cell populations with self-renewal potential that are capable of reconstituting the full diversity of T-cells.

Importantly, a large fraction of mice develop T-ALL in these settings (85). *Tal1* and *Lmo2* expression is strongly upregulated in these mouse T-ALL cells, and *Notch1* mutations are also frequently found. This is consistent with observations in *Tal1* and *Lmo2* transgenic mice, which exhibit LICs in the DN3 subset and acquire *Notch1* mutations (8). One possible mechanism is that differentiation arrest and expansion of DN3 thymocytes caused by overexpression of oncogenic transcription factors result in a loss of competitive replacement by bone marrow-derived progenitor cells, leading to activation of self-renewal machinery and malignant transformation. Alternatively, a loss of competitive replacement may result in the failure to silence the transcription factors that are normally expressed in stem and progenitor cells. Although the intrinsic mechanism of self-renewal in thymocytes is still unclear, these studies suggest that in T-ALL, LICs may arise from thymocytes that already have self-renewal potential *via* cellular competition.

## Transcriptional Regulatory Programs in HSCs and T-Cell Differentiation

Mouse studies have suggested that cellular competition potentially triggers the self-renewal capability of immature thymocytes, which may eventually lead to malignant transformation *via* the acquisition of genetic abnormalities such as *Notch1* mutations. In human T-ALL, a loss of competition may be caused by overexpression of oncogenic transcription factors such as *TAL1* and *LMO2*. Notably, these transcription factors themselves are also involved in the stem cell regulatory program during normal hematopoiesis.

In general, cellular differentiation of hematopoietic cells is associated with developmental restrictions that can be illustrated by the analogy of a “ball rolling down a hill” (86). During the differentiation process, HSCs lose their self-renewal and lineage potential. This process is regulated by an epigenetic and transcriptional network (87–89). A number of hematopoietic transcription factors are involved in this process. For example, TAL1 has been implicated as an essential regulator of hematopoiesis (33). TAL1 is expressed in normal HSCs, progenitor cells, and erythro-megakaryocytic lineages. Studies in knockout mouse models have revealed that this factor is required for hematopoietic specification and the genesis of hematopoietic cells (28, 29). In normal hematopoietic cells, TAL1 forms a large transcriptional complex with E-protein, LMO2, LDB1, and GATA (90). Several other transcription factors, including RUNX1 and the ETS family proteins, also frequently co-regulate downstream target genes (91).

Interestingly, these transcription factors co-occupy their own regulatory elements and positively regulate each other, thus forming an interconnected auto-regulatory loop (87, 88, 92). This structure is also termed a “core regulatory circuit” (CRC) and has been reported in other stem cells (93–95). For example, in embryonic stem cells, three key transcription factors that establish stem cell identity, OCT4, SOX2, and NANOG regulate each other (93, 94). This mechanism is thought to reinforce and stabilize downstream gene expression by “interlocking” the regulatory loop and is likely required for stem cell properties (92). Importantly, ectopic expression of these transcription factors can reprogram somatic cells back into stem cells, as has been established for the production of induced pluripotent stem cells (96). Similarly, recent studies have demonstrated that adult somatic fibroblasts can be reprogrammed into multi-potent hematopoietic stem progenitor cells by ectopic overexpression of *TAL1, LMO2, RUNX1, GATA2*, and *ERG* (“iHSPCs”) (97). This clearly indicates that a relatively small number of transcription factors are sufficient to control cell fate and identity.

**Figure 1:**
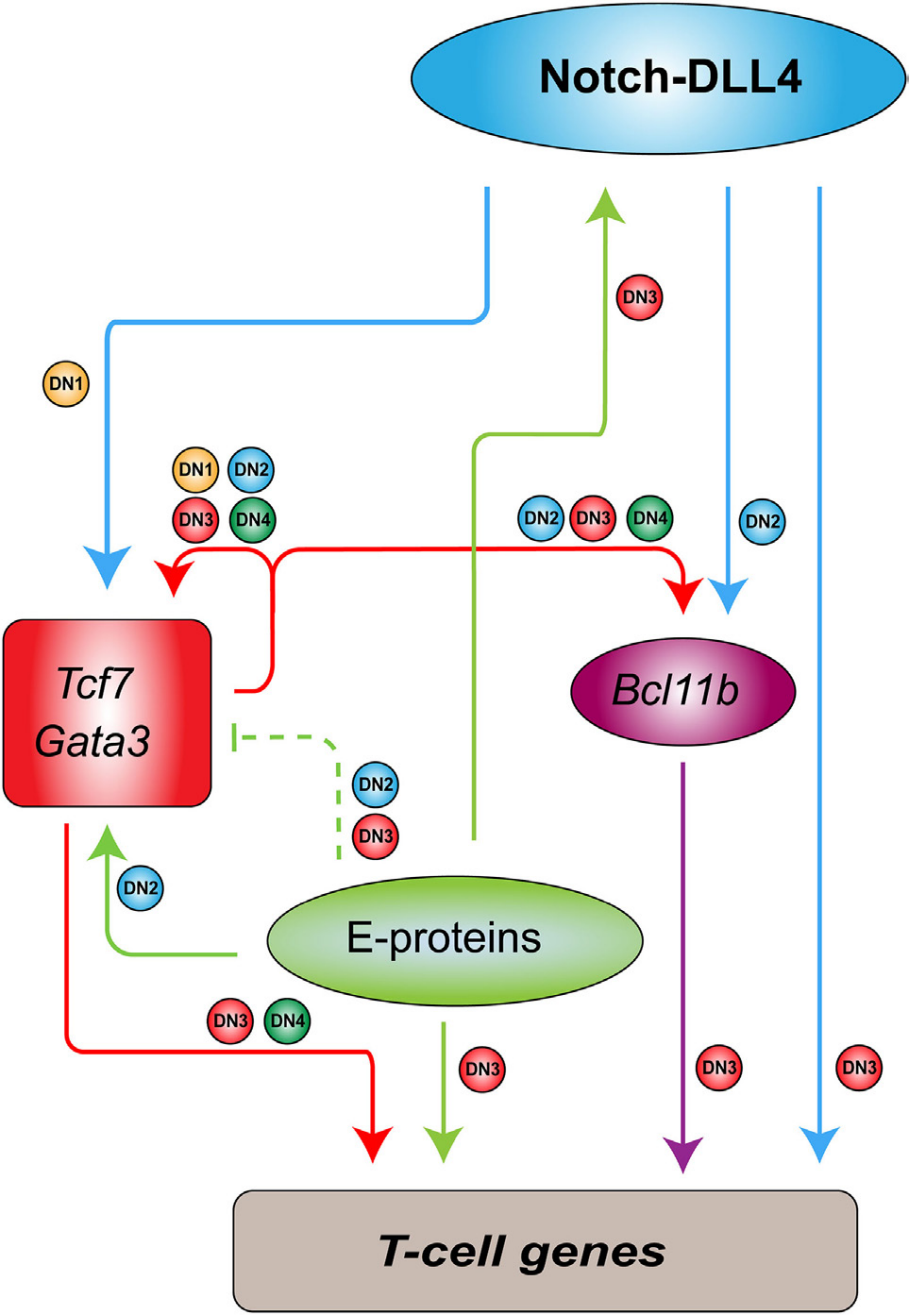
Transcriptional regulatory program in developing thymocytes [adapted and modified from figures by Yui and Rothenberg (98)]. In mouse models, Notch-DLL4 ligand expressed on thymic stroma cells induces the expressions of Tcf7 and Gata3, which regulate additional transcription factors such as Bcl11b. These factors, together with E-proteins and Notch1, stimulate the expressions of T-cell genes in a differentiation stage-specific manner. Arrows show activation or positive regulation. Dashed lines indicate “soft repression” of the maximal activity of the target. Small circles beside the lines correspond to the differentiation stages [double-negative (DN1–4)] at which the regulation occurs.

In contrast to the regulatory circuit in HSCs, a very different type of transcriptional program is formed in developing thymocytes to regulate genes that are essential for T-cell differentiation (98). This process requires a number of transcription factors working in a cascade as well as the interactions in the microenvironment (Figure 1). Briefly, the NOTCH ligand expressed on thymic stromal cells induces expression of the transcription factors TCF7 and GATA3, which regulate other key transcription factors such as BCL11B and LEF1 (98). During this process, stem cell transcription factors such as TAL1 and LMO2 are gradually silenced, resulting in the loss of stem and progenitor cell potential. Meanwhile, E-proteins (E2A and HEB) are functionally and transcriptionally upregulated to induce *RAG1, RAG2*, and *PTCRA*, for example, which are required for somatic *TCR* recombination (99, 100). Such orchestrated stage-specific regulation of transcription factors mediates the T-cell differentiation process like a “ball rolling down a hill.” TAL1 and LMO2 silencing and E-protein upregulation are crucial to controlling the reciprocal switch from self-renewal to lineage-specific genetic programs. In other words, ectopic expression of TAL1 and LMO2 in developing thymocytes may rewrite the internal regulatory program.

## Aberrant Transcriptional Regulatory Program in *TAL1/LMO*-Positive T-ALL

**Figure 2:**
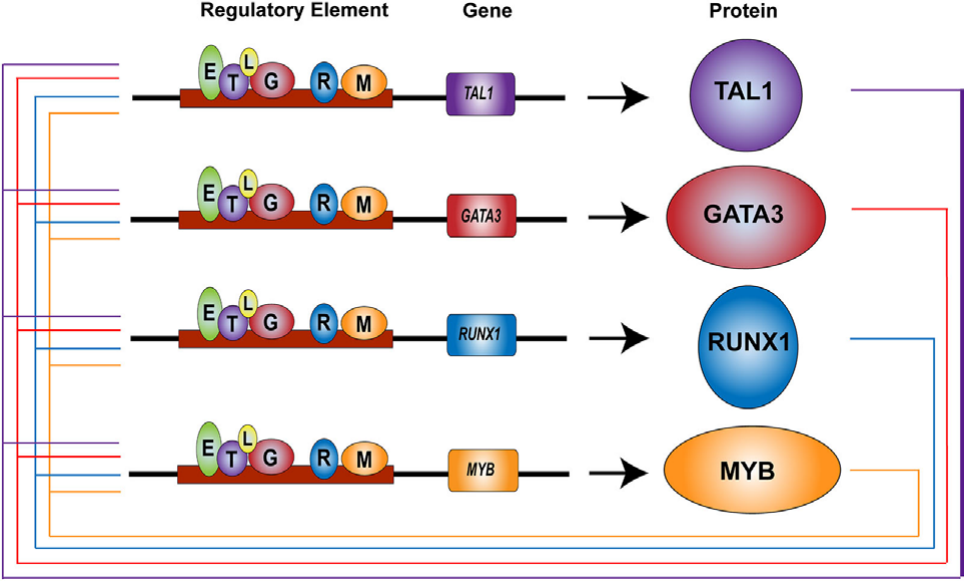
Core regulatory circuit in T-cell acute lymphoblastic leukemia (T-ALL) (38, 105). TAL1, GATA3, RUNX1, and MYB proteins (circles) bind at their own regulatory elements (boxes) and positively regulate each other, thus forming an interconnected auto-regulatory loop structure in T-ALL cells. TAL1 (T), GATA3 (G), RUNX1 (R), MYB (M), E-protein (E), LMO1/2 (L).

Interestingly, *TAL1* and *LMO2* function as oncogenes in T-ALL cells, similar to their behavior in normal HSCs (33). *TAL1* is expressed in 40–60% of T-ALL cases due to chromosomal translocation, intrachromosomal rearrangement, or mutations in non-coding elements (16, 36–38). These alterations replace an endogenous regulatory element controlling *TAL1* expression with a new, potent enhancer that drives ectopic expression of this oncogene. Similarly, *LMO2* or its related gene *LMO1* is ectopically expressed in T-ALL cells due to chromosomal translocation or mutations in the regulatory elements (16, 101, 102). *LMO1* or *LMO2* is often expressed together with *TAL1*. In T-ALL cells, TAL1 and LMO proteins form a transcriptional complex with E-proteins and GATA3 (103, 104). Their regulatory partners in normal HSCs, RUNX1, ETS1, and MYB are also endogenously expressed in T-cells (98). We previously reported that TAL1, GATA3, RUNX1, and MYB co-occupy their own regulatory elements and positively regulate each other, forming the interconnected auto-regulatory structure (Figure 2) (105). These factors coordinately regulate downstream target genes. All these mechanisms are essentially the same as the machinery observed in normal HSCs.

**Figure 3:**
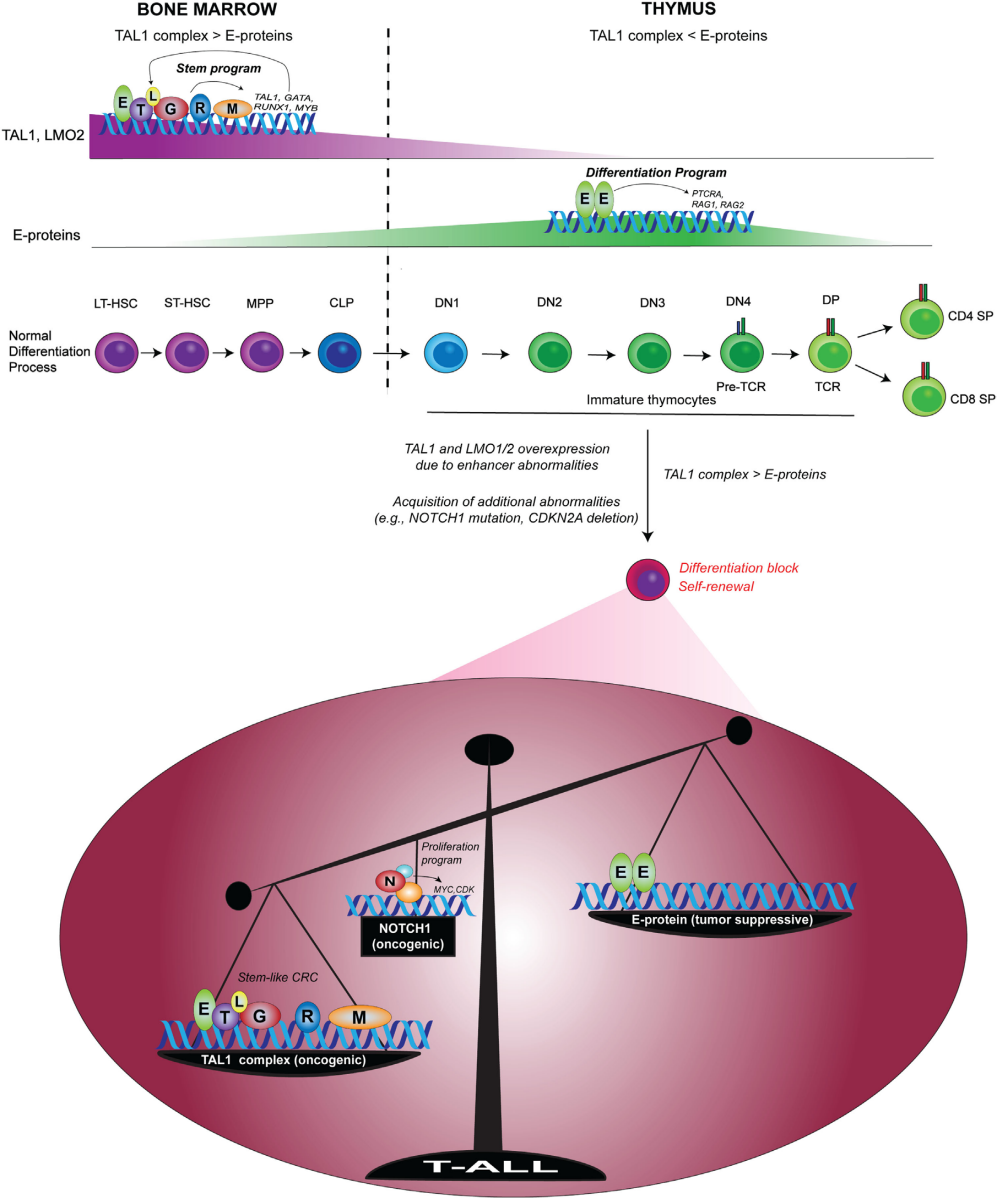
Imbalance between the oncogenic TAL1 complex and E-protein tumor suppressor in T-cell acute lymphoblastic leukemia (T-ALL) [modified from a figure by Sanda and Leong (107)]. In normal hematopoiesis, TAL1 forms a transcriptional complex with E-protein, GATA2, RUNX1, MYB, and LMO2 to drive a regulatory program in HSCs and progenitor cells *via* the auto-regulatory loop. Upon the progression of T-cell commitment in the thymus, TAL1 and LMO2 expressions are silenced, while E-proteins are functionally and transcriptionally upregulated. E-protein dimers induce the expressions of *RAG1, RAG2*, and *PTRCA* to prompt the differentiation program of T-cells. In T-ALL, enhancer abnormalities (chromosomal translocation, intrachromosomal rearrangement or mutations in the enhancer) cause ectopic expressions of *TAL1* and/or *LMO1/2*, leading to the formation of TAL1 complex and the inhibition of E-protein dimers. T-ALL cells also acquire additional abnormalities such as genetic mutations of *NOTCH1* and deletion of *CDKN2A*. TAL1 and its regulatory partners form a stem cell-like core regulatory circuit (CRC) and NOTCH1 activates a different set of genes such as *MYC*. The functional imbalance between the oncogenic TAL1 complex and E-protein tumor suppressors possibly contributes to the induction of self-renewal program and the blockade of T-cell differentiation machinery. Mutated NOTCH1 boosts this oncogenic mechanism. LT-HSC, long-term HSC; ST-HSC, short-term HSC; MPP, multipotent progenitor; CLP, common lymphoid progenitor; DN, CD4^−^CD8^−^ double-negative; DP, CD4^+^CD8^+^ double-positive; SP, CD4^+^ or CD8^+^ single-positive; T, TAL1; E, E-protein; L, LMO1/2; G, GATA; R, RUNX1; M, MYB; N, NOTCH1.

At the same time, TAL1 counteracts the function of E-proteins by sequestering them, thus preventing them from transcriptionally inducing genes required for T-cell differentiation (99, 100). In this context, E-proteins act as tumor suppressors, as several groups have shown that *E2a*-deficient mice develop T-cell lymphoma and that this deficiency accelerates leukemia onset and progression in *Tal1*-transgenic mice (10, 106). Our recent study also revealed that in human T-ALL cells, TAL1 opposes the expression of E-protein target genes (105). Thus, the imbalance between the oncogenic TAL1 complex and E-protein is a primary determinant underlying the molecular pathogenesis of T-ALL (Figure 3) (107). Together, ectopic expression of *TAL1* and *LMO1/2* leads to the induction of HSC-like machinery and disruption of the T-cell differentiation program.

## Potential Stem Cell Signature Induced by TAL1 in T-ALL

In this regard, it would be interesting to identify genes that are abnormally induced by the TAL1 complex in T-ALL cells. Recently, our group used a targeted approach to identify regulatory elements that are differentially controlled by TAL1 and E-proteins (108). From this analysis, we discovered an enhancer situated within a cluster of seven genes belonging to the *GTPase of Immunity Associated Protein* (*GIMAP*) family. This region is associated with active histone marks in T-ALL cells but not in the normal human thymus, suggesting that the *GIMAP* enhancer is aberrantly activated in T-ALL cells. Importantly, *GIMAP* genes are expressed in mouse HSCs and CD4 or CD8 SP mature T-cells, while they are downregulated in DN3-4 stage thymocytes where TAL1 is also silenced. Using an *in vivo* reporter system in zebrafish, we showed that the *GIMAP* enhancer can be activated in normal hematopoietic stem and progenitor cells but not in the thymus. In addition, a reporter assay in human T-ALL cell lines indicated that the *GIMAP* enhancer is activated by TAL1 and its regulatory partners (GATA3 and RUNX1) and is repressed by E-proteins (E2A and HEB). Although ectopic expression of human *GIMAP* genes in immature zebrafish thymocytes did not induce tumor formation, their overexpression accelerated leukemia development in the presence of the *MYC* oncogene. Thus, our results revealed that aberrant activation of the *GIMAP* enhancer contributes to T-cell leukemogenesis.

While *GIMAP* genes have been known to be involved in the development of mature T- and B-lymphocytes (109–112), another group has also implicated their importance in HSC survival and maintenance (113). The work of Chen et al. on *Gimap5*^−^*^/^*^−^ mice demonstrated that Gimap5 regulates the survival of HSCs and other early hematopoietic progenitors by stabilizing the Mcl-1 protein, which is an anti-apoptotic Bcl-2 family member (113). The HSCs in *Gimap5*-deficient mice exhibited defective long-term repopulation capacity, as demonstrated by their impaired engrafting ability. This study provided insights into the critical roles of *GIMAP* genes in the survival of HSCs and early progenitor cells. Notably, NOTCH1 was also identified as a positive regulator of the *GIMAP* genes in T-ALL cells (114, 115). A functional study by Chadwick et al. showed that *Gimap5* mediates apoptosis protection in T-ALL cells upon its upregulation by NOTCH1 (114). Together with our findings, these studies suggest that as a consequence of *TAL1/LMO* overexpression and activation of the NOTCH1 pathway, the *GIMAP* genes could be reactivated in immature thymocytes in which they are normally repressed, possibly by E-proteins, thereby contributing to leukemogenesis.

Another gene that has been implicated in stem cells and is also aberrantly activated by the TAL1 complex in T-ALL is the *ALDH1A2* gene (105, 116). Based on our ChIP-seq and gene expression data, this gene was one of the top candidate genes directly regulated by TAL1 in human T-ALL cells (105). ALDH activity has been proposed to be a universal CSC marker, as demonstrated by the tumorigenic and self-renewal properties of ALDH^+^ cells isolated from leukemia and many solid tumors (117–119). Among the 19 isoforms in the ALDH family, only a few of them, including *ALDH1A2* are involved in retinoic acid signaling, which has been known to be associated with the stemness characteristics of CSCs. Another group and our recent study indicated that *ALDH1A2* is induced by TAL1 *via* an internal enhancer in T-ALL cells (116) (and Zhang and Tan et al., unpublished data). Although the role of *GIMAPs* and *ALDH1A2* in the self-renewal potential of malignant T-cells is yet to be elucidated, their ability to mark stem cells and T-ALL cells may be used as a signature of the aberrant transcriptional program induced by T-ALL oncogenes.

## Conclusion and Future Prospective

The transformation mechanism in T-ALL is very efficient. T-ALL oncogenes alter the intrinsic transcriptional regulatory program by disrupting the differentiation machinery and by introducing the stem cell-like properties into developing thymocytes. This may initiate or reactivate the self-renewal ability that potentially exists in thymocytes. This process is mediated by a relatively small number of oncogenic transcription factors and seems not require the accumulation of a large number of genetic and chromosomal abnormalities until it obtains the hallmarks of cancer.

In other words, this mechanism poses a potential severe risk hidden in the thymus. Thymocytes may always be “primed” to initiate leukemogenesis. As recently reported (17, 18, 84), the competitive replacement of thymocytes *via* a continuous supply of lymphoid progenitor cells from the bone marrow plays an important tumor suppressive role in homeostasis. Further investigation is necessary to elucidate the loss-of-competition mechanism in human T-ALL. In particular, it is of great interest to analyze whether T-ALL develops from a self-renewal pool prior to the *TCR* rearrangement or pre-leukemic clones, which harbor the *TCR* translocation newly acquire the self-renewal capability. Single cell sequencing analysis is ideal to detect the emergence of those clones. Another important consideration is the mechanism of self-renewal in the ETP subtype of T-ALL. ETP cases show a very different genomic landscape and gene expression signature as compared to non-ETP cases. For example, mutations of *NOTCH1* are less frequently found in ETP (27), thus suggesting that different oncogenic mechanisms are involved. Establishment of proper model systems is needed to analyze LICs in this particular subtype.

The mechanisms described above can be also therapeutic targets to eliminate LICs in T-ALL. Disruption of the transcriptional complex involving TAL1 would efficiently block the formation of the CRC and revert the functional imbalance between oncogenic TAL1 complex and E-protein tumor suppressors. Rabbit and his colleagues have developed a peptide and intracellular antibody targeting LMO2 protein to disassociate the TAL1–LMO2 complex (32, 120, 121). Inhibition of transcriptional machinery by small-molecule inhibitors of CDK7 or BRD4 concurrently reduces expressions of multiple oncogenic transcription factors in T-ALL, thereby leading to cell death (122, 123). Moreover, targeting CXCR4/CXCL12 signaling is an ideal strategy to disrupt the interaction between T-ALL cells and stroma cells in the bone marrow niche, as recently reported (82, 83). Additionally, identification of specific cell surface markers associated with LIC capability in T-ALL is critical for developing better therapeutic strategy.

## Author Contributions

All authors listed have made a substantial, direct, and intellectual contribution to the work and approved it for publication.

## Conflict of Interest Statement

All authors declare that the research was conducted in the absence of any commercial or financial relationships that could be construed as a potential conflict of interest.
